# A revision of the genus *Elimedon* J.L. Barnard, 1962 (Crustacea, Amphipoda, Tryphosidae) with the addition of two new species from the Clarion-Clipperton Zone, Central Pacific Ocean

**DOI:** 10.3897/zookeys.1274.139884

**Published:** 2026-03-24

**Authors:** Tammy Horton, Georgina Valls Domedel, Eva C. D. Stewart, Ed A. Hendrycks

**Affiliations:** 1 National Oceanography Centre, Southampton, SO14 3ZH, UK National Oceanography Centre Southampton United Kingdom; 2 School of Ocean and Earth Sciences, University of Southampton, Southampton, SO14 3ZH, UK University of Southampton Southampton United Kingdom; 3 Life Sciences Department, Natural History Museum, London, Cromwell Road, South Kensington, SW7 5BD, UK Canadian Museum of Nature, Research Associate, Research and Collections Ottawa Canada; 4 Canadian Museum of Nature, Research Associate, Research and Collections, P.O. Box 3443, Station D, Ottawa, K1P 6P4, Canada Life Sciences Department, Natural History Museum London United Kingdom

**Keywords:** Abyss, amphipods, Clarion-Clipperton Zone, deep-sea, Lysianassoidea Pacific Ocean

## Abstract

Two new species are described in the genus *Elimedon* within the family Tryphosidae from depths between 4026–4299 m in the Clarion-Clipperton Zone, Pacific Ocean. A revised diagnosis of *Elimedon* is provided clarifying the characters which distinguish the genus from the closely related *Hippomedon* and allied genera. *Elimedon breviclunis***sp. nov**. is described from eight specimens collected between 4273–4299 m depth and is characterised by a strongly shortened uropod 3 inner ramus and a telson which is long and deeply cleft, with widely separated lobes. *Elimedon zabka***sp. nov**. is described from a single specimen of unknown sex collected at 4026 m depth. This species is characterised by a strongly shortened uropod 3 inner ramus, and a shortened (length equal to width) truncate telson. Molecular barcode data are also provided for the two species. The new species are fully illustrated and a key to the four species of *Elimedon* is provided.

## Introduction

The genus *Elimedon* J.L. Barnard, 1962 has until now contained just two species. It was originally described for the species *Elimedon cristatus* J.L. Barnard, 1962, based on a single specimen collected in the South Atlantic Ocean, Angola Basin at 3916 m. A second species was referred to the genus *Elimedon* by Barnard and Karaman (1991) when they transferred Ledoyer’s species, *Hippomedon brevicaudatus* (Ledoyer, 1986), a single specimen collected from the Indian Ocean, Madagascar plateau at 3923–3933 m. Barnard (1962) stated that *Elimedon* could be distinguished from the closely related *Hippomedon* Boeck, 1871 only by the “short third palp article of the mandible and the short second antenna” (Barnard 1962). Barnard (1964, 1969) later provisionally synonymised both *Paracentromedon* Chevreux & Fage, 1925, and *Elimedon* with *Hippomedon*. Jarrett and Bousfield (1982) provided updated diagnoses and a key to *Hippomedon* and allied genera (*Paracentromedon*, *Paratryphosites* Stebbing, 1899, *Psammonyx* Bousfield, 1973 and *Wecomedon* Jarrett & Bousfield, 1982). Barnard and Karaman (1991) retained the allied genera separate from *Hippomedon*. The genus *Wecomedon* was synonymised with *Psammonyx* by Budnikova (2005), and it was later recognised that *Psammonyx* was a junior homonym of *Psammonyx* Döderlein, 1892 (Foraminifera), so the name was replaced by a revived *Wecomedon* (see Horton 2018). The characters separating these allied genera and the large and diverse genus *Hippomedon* are in need of further study, which is beyond the scope of this paper.

Here we add two new species to the genus *Elimedon* from the Clarion-Clipperton Zone, which extends the geographic distribution of the genus from the south Atlantic and Indian Ocean to the Pacific Ocean and the depth range from 3916 to 4298 m. The addition of these two new species to this rarely collected genus has also allowed us to provide an amended diagnosis of *Elimedon* to further clarify the characters which separate it from the closely related, large and diverse genus *Hippomedon*. Both new species are illustrated and a key to *Elimedon* species is given. We also provide molecular barcode sequences for both species which will enable further understanding of the genus and the relationships within the Tryphosidae.

## Materials and methods

Material for the present study was sampled in the central-east Pacific Ocean, specifically in the easternmost sector of Clarion-Clipperton Zone (CCZ). The material was collected using either an epibenthic sledge (EBS) or a USNEL Spade Box Core (Ocean Instruments BX-650; BC) during four expeditions to two different exploration contract areas (henceforth, contract areas) in the CCZ: the UKSR-1 contract area (ABYSSLINE-2; ABYSSal baseLINE project; Smith et al. 2015), and three cruises to the NORI-D region (C5A in 2020; C5D in 2021; and C7A in 2022) following methods in Glover et al. (2015). For details of gear types and sample processing see the relevant cruise reports and Jażdżewska and Horton (2026).

The habitus of the holotype specimen of *Elimedon zabka* sp. nov. is presented as a photograph obtained with a confocal laser scanning microscope (CLSM). The specimen was stained in Congo red and acid fuchsin, temporarily mounted onto slides in glycerol and examined with a Leica TCS SPV equipped with a Leica DM5000 B upright microscope and three visible-light lasers (DPSS 10 mW 561 nm; HeNe 10 mW 633 nm; Ar 100 mW 458, 476, 488 and 514 nm), combined with the software LAS AF 2.2.1 (Leica Application Suite, Advanced Fluorescence). A series of photographic stacks were obtained, collecting overlapping optical sections throughout the whole preparation (Michels and Büntzow 2010; Kamanli et al. 2017).

Holotype specimens were dissected and mounted onto permanent slides using polyvinyl-lactophenol stained with lignin pink. Illustrations were made using Nikon SMZ1500, Nikon Eclipse Ci, Leica M125 or Olympus BX53 microscopes equipped with a camera lucida. Pencil drawings were scanned and inked digitally using Adobe Illustrator and a WACOM digitiser tablet (Coleman 2003, 2009). Some setae are omitted from the illustrations for clarity. Appendages of the right side are dissected and illustrated, unless otherwise stated.

In the descriptions and figures the following abbreviations were used: **A1, A2** = antenna 1, 2; **c1–c4** = coxa 1–4; **G1, G2** = gnathopod 1, 2; **LL** = lower lip; **Md** = mandible; **Mx1, Mx2** = maxilla 1, 2; **Mxp** = maxilliped; **P3–P7** = pereopod 3–7; **U1–U3** = uropod 1–3; **UL** = upper lip; **T** = telson; **l** = left; **r** = right.

Type material is deposited in the Senckenberg Museum (Frankfurt, Germany) (**SMF**), the Natural History Museum, London (**NHMUK**), and the Canadian Museum of Nature, Ottawa (**CMNC**).

### DNA extraction, amplification, and sequencing

Specimens collected from the ABYSSLINE-2 cruise were extracted and sequenced as described in Jażdżewskaet al. (2025). Specimens collected from the NORI-D area were processed as follows. DNA was extracted from a pair of pleopods using QuickExtract^TM^ DNA extraction solution (Lucigen), following manufacturer guidelines, and adapted for a digestion time of 45 min. Regions of two mitochondrial [16S rRNA (16S) and cytochrome oxidase subunit I (COI)] and three nuclear [28S rRNA (28S), 18S rRNA (18S), and early-stage histone 3 (H3)] genetic markers were amplified with published primer sets (Medlin et al. 1988; Nygren and Sundberg 2003; Astrin and Stüben 2008; Corrigan et al. 2014; Lörz et al. 2018). The PCR mix for each reaction contained 10.5 µl of Red Taq DNA Polymerase 1.1X MasterMix (VWR), 0.5 µl of each primer (10 µM), and 1 µl of DNA template. Primers and PCR conditions are detailed in Table 1. The primers used for sequencing were the same as those for amplifications, with an additional set of internal primers for 18S: 620F (TAAAGYTGYTGCAGTTAAA; Nygren and Sundberg 2003) and 1324R (CGGCCATGCACCACC; Cohen et al. 1998). PCR products were purified using a Millipore Multiscreen 96-well PCR Purification System and sequenced using an ABI 3730XL DNA Analyzer (Applied Biosystems) at The Natural History Museum Sequencing Facilities. For each gene fragment contigs were assembled by aligning both forward and reverse sequences, chromatograms were visually inspected, and ambiguous base calls were corrected manually, using Geneious 7.0.6 (Kearse et al. 2012). Sequence divergences were compared within and between the *Elimedon* species using the Kimura two-parameter (K2P) distance model (Kimura 1980) implemented with MEGA X, using five COI sequences (four of *E. breviclunis* and one of *E. zabka*). The relevant voucher information, taxonomic classifications and sequences are deposited in the data set “DS-AMPHICCZ” in the Barcode of Life Data System (BOLD) (https://doi.org/10.5883/DS-AMPHICCZ) (www.boldsystems.org) (Ratnasingham and Hebert 2007).

**Table 1.** Primers and PCR programs used for DNA amplification.

**Gene**	**Primer**		**Sequence (5’ – 3’)**	**PCR program**	**Reference**
COI	LCO1490-JJ	Forward	CHACWAAYCATAAAGATATYGG	1 × (2 min at 94 °C), 5 × (30 s at 94 °C, 90 s at 45 °C, 60 s at 72 °C), 35 × (30 s at 94 °C, 90 s at 51 °C, 60 s at 72 °C), 1 × (5 min at 74 °C)	Astrin and Stüben 2008
	HCO2198-JJ	Reverse	AWACTTCVGGRTGVCCAAARAATCA		Astrin and Stüben 2008
16S	16SFt_amp	Forward	GCRGTATIYTR ACYGTGCTAAGG	1 × (2 min at 95 °C), 35 × (30 s at 95 °C, 30 s at 50 °C, 45 s at 72 °C), 1 × (5 min at 72 °C)	Lörz et al. 2018
	16SRt_amp	Reverse	CTGGCTTAAACCGRTYTGAACTC		Lörz et al. 2018
28S	28Sftw	Forward	AGGCGGAATGTTGCGT	1 × (2 min at 95 °C), 35 × (40 s at 94 °C, 40 s at 50 °C, 40 s at 72 °C), 1 × (10 min at 72 °C)	Corrigan et al. 2014
	28Srtw	Reverse	CTGAGCGGTTTCACGGTC		Corrigan et al. 2014
18S	18SA	Forward	AYCTGGTTGATCCTGCCAGT	1 × (5 min at 95 °C), 30 × (30 s at 95 °C, 30 s at 59 °C, 60 s at 72 °C), 1 × (10 min at 72 °C)	Medlin et al. 1988
	18SB	Reverse	ACCTTGTTACGACTTTTACTTCCTC		Nygren and Sundberg 2003
H3	HisH3f	Forward	AAATAGCYCGTACYAAGCAGAC	1 × (2 min at 95 °C), 35 × (40 s at 94 °C, 40 s at 45 °C, 40 s at 72 °C), 1 × (10 min at 72 °C)	Corrigan et al. 2014
	HisH3r	Reverse	ATTGAATRTCYTTGGGCATGAT		Corrigan et al. 2014

## Results

### Systematics

**Order AMPHIPODA Latreille, 1816**

**Suborder AMPHILOCHIDEA Boeck, 1871**

**Superfamily LYSIANASSOIDEA Dana, 1849**

**Family TRYPHOSIDAE Lowry & Stoddart, 1997**

#### Genus Elimedon

Taxon classificationAnimaliaAmphipodaTryphosidae

J.L. Barnard, 1962

07614777-4607-5799-BAE6-E90392AF5DBA

Elimedon J.L. Barnard, 1962: 24. ―J.L. Barnard 1964: 5. ―J.L. Barnard 1969: 345. ―Jarrett and Bousfield 1982: 105. ―Ledoyer 1986: 751. ― Barnard and Karaman 1991: 483.

##### Type species.

*Elimedon cristatus* J.L. Barnard, 1962 (original designation).

##### Included species.

*Elimedon* contains four species: *Elimedon brevicaudatus* (Ledoyer, 1986); *E. breviclunis* sp. nov.; *E. cristatus* J.L. Barnard, 1962; *E. zabka* sp. nov.

##### Diagnosis

(modified in italics after Barnard and Karaman 1991). Mouthparts forming quadrate bundle. Labrum and epistome not prominent, separate, labrum slightly dominant in projection, blunt. Incisor ordinary; molar triturative, large; palp attached opposite molar. Inner plate of maxilla 1 moderately (three) setose; palp bi-articulate, large. Inner and outer plates of maxilliped well developed, palp scarcely exceeding outer plate, dactyl well developed. Coxa 1 large and visible, not tapering. Gnathopod 1 short, strongly subchelate, palm acute, carpus longer than propodus, dactyl large; gnathopod 2 propodus much shorter than carpus, ordinary, propodus ***minutely subchelate***. Inner ramus of uropod 2 without notch. Uropod 3 aequiramous ***or strongly reduced***, peduncle ordinary, outer ramus bi-articulate. Telson elongated ***or shortened***, ***cleft 50%***.

##### Remarks.

According to Barnard and Karaman (1991) the genus *Elimedon* differs from *Hippomedon*, (and *Wecomedon*) in possessing a shortened article 3 of the mandibular palp (half or less the length of article 2, compared to *Hippomedon* which has articles 2 and 3 approximately subequal). In addition, the bases of pereopods 5 and 6 are narrow in *Elimedon* versus broad in *Hippomedon*; and *Elimedon* lacks a gill on pereopod 7, while possession of a reduced gill on pereopod 7 is an important character for *Hippomedon* (Jarrett and Bousfield 1982). The short antenna 2 of the female (~ 1.5 times as long as antenna 1) is also diagnostic for *Elimedon*. In *Hippomedon*, antenna 2 is elongate in both sexes.

*Elimedon* can be separated from the closely allied *Paracentromedon* in the telson being ~ 50% cleft in *Elimedon* (versus ~ 75% in *Paracentromedon*), and in the gnathopod 1 characters. In *Paracentromedon* the gnathopod 1 is almost simple with the carpus and propodus subequal, compared to the strongly subchelate gnathopod 1 of *Elimedon* with the carpus longer than propodus.

#### Elimedon breviclunis sp. nov.

Taxon classificationAnimaliaAmphipodaTryphosidae

1924EBF2-D206-5BB5-A192-2E2E2BB32A2A

https://zoobank.org/72B51C3C-DD7B-4279-AF2A-BD24ACD6A6DB

Figs 1, 2, 3, 4, 5

##### Type material.

***Holotype***: Pacific • female (with non-setose oostegites), 12.8 mm (dissected carcass); carcass and eight slides; Clarion-Clipperton Zone; 10.5313°N, 117.304°W; depth 4298 m; 13/11/2020; NORI-D contract area, RV "Maersk Launcher", Cruise 5A, Station SWM_022, Box Core BC_359; Specimen 5999_TH_AMP1; NHMUK 2025.10, COI (PV077112), 16S (PV077021). ***Paratypes***: Pacific • immature (male), 9.2 mm; Clarion-Clipperton Zone; 10.359°N, 117.2405°W; depth 4298 m; 19/05/2021; NORI-D contract area, RV "Maersk Launcher", Cruise 5D, Station STM_190, Box Core BC_407; Specimen 7969_TH_AMP1; NHMUK 2025.11. • male, 10.7 mm; Clarion-Clipperton Zone; 10.3333°N, 117.192°W; depth 4287 m; 31/05/2021; NORI-D contract area, RV "Maersk Launcher", Cruise 5D, Station STM_196, Box Core BC_419; Specimen 8595_TH_AMP_1; CMNC 2025-0002. • immature (damaged), 4.8 mm; Clarion-Clipperton Zone; 10.3559°N, 117.169°W; depth 4277 m; 11/11/2020; NORI-D contract area, RV "Maersk Launcher", Cruise 5A, Station STM_010, Box Core BC_355; Specimen 8802_TH_AMP1; NHMUK 2025.12, COI (PV077022) 28S (PV077026), H3 (PV078006). • immature, 6.5 mm; Clarion-Clipperton Zone; 10.3277°N, 117.184°W; depth 4284 m; 25/08/2022; NORI-D contract area, MV Island Pride, Cruise 7A, Station TF_004, Box Core BC_430; Specimen 9350_TH_AMP3; NHMUK 2025.13, COI (PV077115), 16S (PV077024), 28S (PV077028), H3 (PV078008).

##### Other material.

Pacific • unsexed (urosome only), not measured; Clarion-Clipperton Zone; 10.3559°N, 117.169°W; depth 4277 m; 11/11/2020; NORI-D contract area RV "Maersk Launcher", Cruise 5A, Station STM_010, Box Core BC_355; Specimen 8803_TH_AMP_1; NHMUK 2025.15. • immature (damaged), not measured; Clarion-Clipperton Zone; 10.3341°N, 117.185°W; depth 4284 m; 29/05/2021; NORI-D contract area, RV "Maersk Launcher", Cruise 5D, Station STM_199, Box Core BC_416; Specimen 8581_TH_AMP1; NHMUK 2025.14, COI (PV077113) • immature, (damaged, not measured); Clarion-Clipperton Zone; 10.3575°N, 117.17°W; depth 4273 m; 09/09/2022; NORI-D contract area, MV "Island Pride", Cruise 7A, Station STM_173, Box Core BC_451; Specimen 9337_TH_AMP2; NHMUK 2025.16, COI (PV077114), 16S (PV077023), 18S (PV077025), 28S (PV077027), H3 (PV078007).

##### Type locality.

Abyssal Pacific Ocean, Clarion-Clipperton Zone, 10.5313°N, 117.304°W; depth 4298 m.

##### Diagnosis.

Mandibular palp article 3 short, 0.7 × length of article 2; gnathopod 1 carpus slightly longer than propodus (1.45×); Pereopods 5 and 6 bases, both narrow, length twice width; uropod 3 inner ramus strongly shortened, length 0.3 × outer ramus; telson long, length 1.5 × width, widely cleft and V-shaped, ~ 52%.

##### Description.

Based on holotype immature female (non-setose oostegites), 12.8 mm, NHMUK 2025.10.

**Figure 1.**
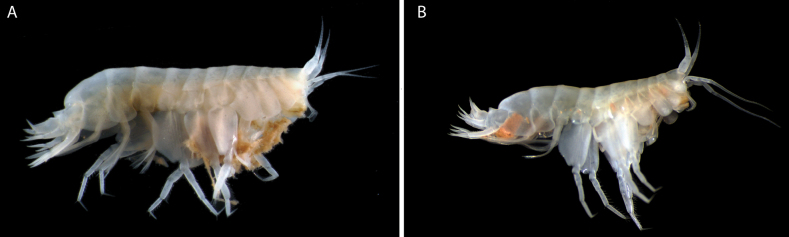
**A** photograph of *Elimedon breviclunis* sp. nov., habitus of holotype immature female, 12.8 mm, NHMUK 2025.10 **B** photograph of *Elimedon breviclunis* sp. nov., habitus of paratype male, 10.7 mm, CMNC 2025-0002.

**Figure 2.**
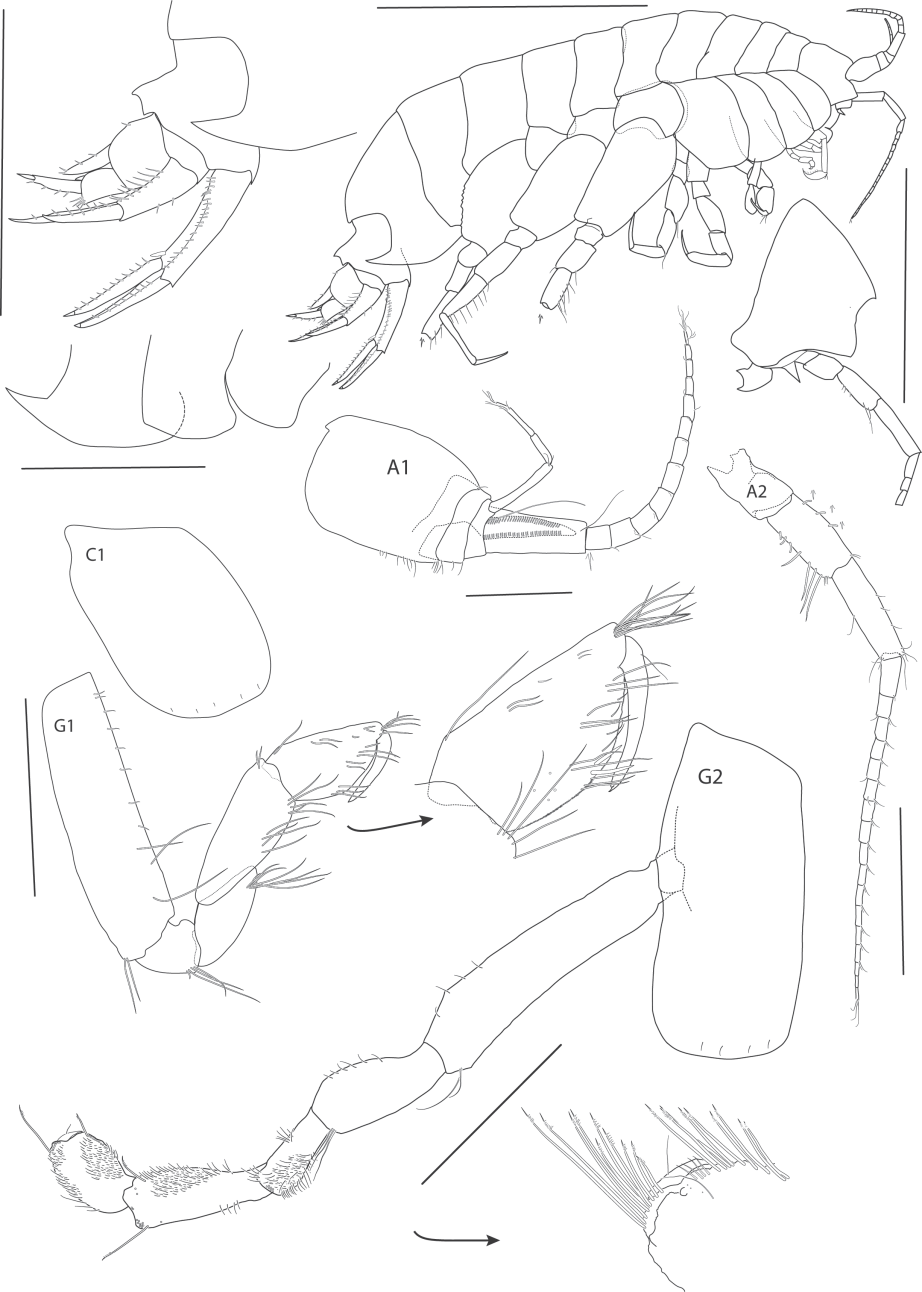
*Elimedon breviclunis* sp. nov., immature female, 12.8 mm. NHMUK 2025.10. Scale bars: 5 mm (habitus); 3 mm (epimeres, urosome, head); 0.5 mm (A1); 1 mm (A2, C1, G1, G2).

**Body** (Figs 1, 2): ***Pereonites 1*–*7*** broader than deep. ***Pleonite 3*** with a rounded, posterodorsal elevation slightly overhanging urosomite 1. ***Urosomite 1*** with a slight dorsal concavity in front of acute carina, carina slightly pointing backward and overhanging urosomite 2. ***Urosomite 2*** very short, telescoped under urosomite 1. ***Epimeron 1*** quadrate, anterodistal corner slightly narrow. ***Epimeron 2*** subquadrate, anterodistal corner rounded, distal margin convex, posterodistal corner not produced, posterior margin straight. ***Epimeron 3*** ventral margin strongly convex, posterodistal corner produced into a long, slightly upturned tooth. ***Coxae 1*–*4*** longer than corresponding pereonites, progressively longer, coxa 1 not shortened.

**Head** (Figs 1, 2): Approximately equal in length to pereonites 1 and 2; rostrum short, not reaching end of lateral cephalic lobe. ***Lateral cephalic lobe*** broadly triangular, subacute. ***Eye*** not present. ***Antenna 1*** short, length 0.2 × body; peduncular article 1 dilated, length 1.3 × width, with slight dorsal keel projecting distally over article 2; peduncular articles 2 and 3 very short; flagellum 13-articulate, first article of flagellum callynophorate, furnished medially with double row of aesthetascs; accessory flagellum 4-articulate, first article slightly broader and equal to remaining articles combined, calceoli absent. ***Antenna 2*** slightly longer than antenna 1, gland cone present (damaged on dissection); peduncular article 4 shorter than 5, with posteromedial setae; flagellum 17-articulate, calceoli absent.

**Figure 3.**
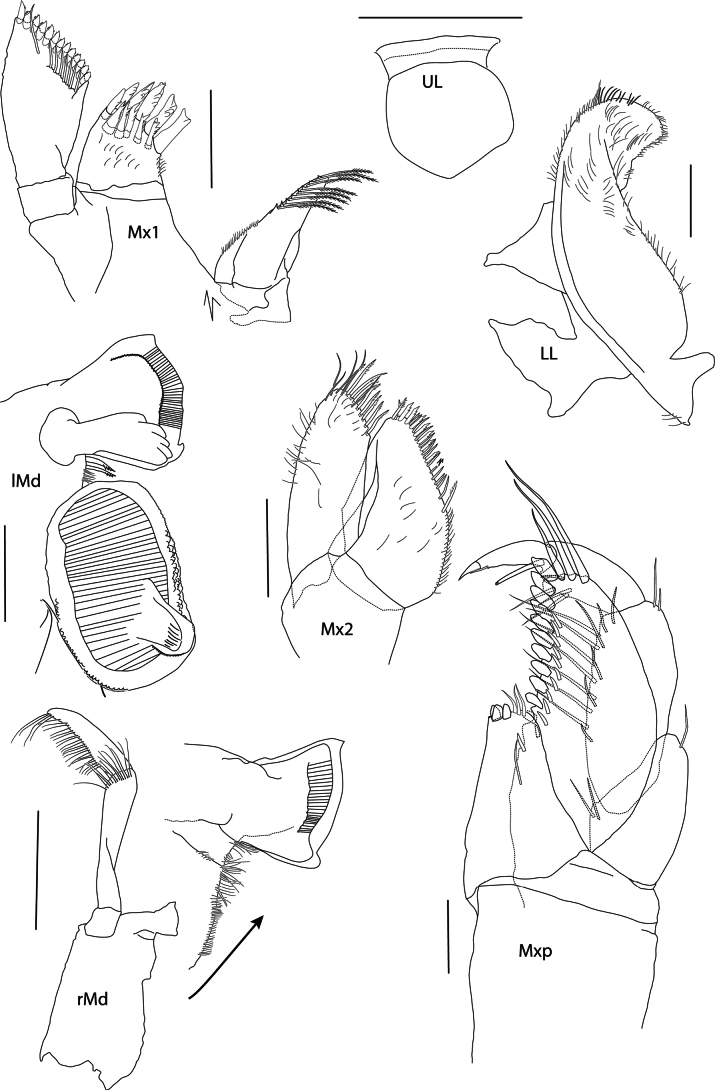
*Elimedon breviclunis* sp. nov., immature female, 12.8 mm, NHMUK 2025.10. Scale bars: 0.2 mm (Mx1, Mx2); 0.5 mm (UL); 0.1 mm (LL, Mxp, lMd); 0.5 mm (Md).

**Mouthparts** (Figs 1, 2, 3): ***Epistome*** straight, smooth, upper lip slightly dominant. ***Upper lip*** (Fig. 2, 3): broadly rounded. ***Mandible*** (Fig. 3): incisor convex and widened, with tooth at anterodistal and posterodistal corners; left lacinia mobilis 5-dentate, right lacking; accessory spine row with two small spines; molar ovate and strongly triturative; palp attached distal to molar, article 2 1.43 × length of article 3, with 14 A2-setae, article 3 falciform, 0.7 × length of article 2, with 22 D3-pectinate setae and four E3-setae. ***Lower lip*** (Fig. 3): damaged. ***Maxilla 1*** (Fig. 3): inner plate narrowing distally, inner margin with eight plumose setae; outer plate broad, with 11 spine-teeth in 6/5 crown arrangement; palp two-articulate, article 2 widened distally, with 13 contiguous conical apical spines, and eight facial setae. ***Maxilla 2*** (Fig. 3): inner plate slightly shorter and broader than inner, tapering distally, both with row of pectinate medial marginal spines and setae. ***Maxilliped*** (Fig. 3): inner plate subrectangular, extending slightly past the distal end of the inner margin of palp article 1 and not reaching one-half of outer plate, distal margin with three nodular spines and three setae; outer plate narrowly subovate, length 2.15 × width, extending past the distal end of palp article 2, with three strong distal setae and 11 strong medial nodular spines, medial margin very slightly convex; palp setose medially, article 2 longest, article 4 slightly shorter than article 3.

**Figure 4.**
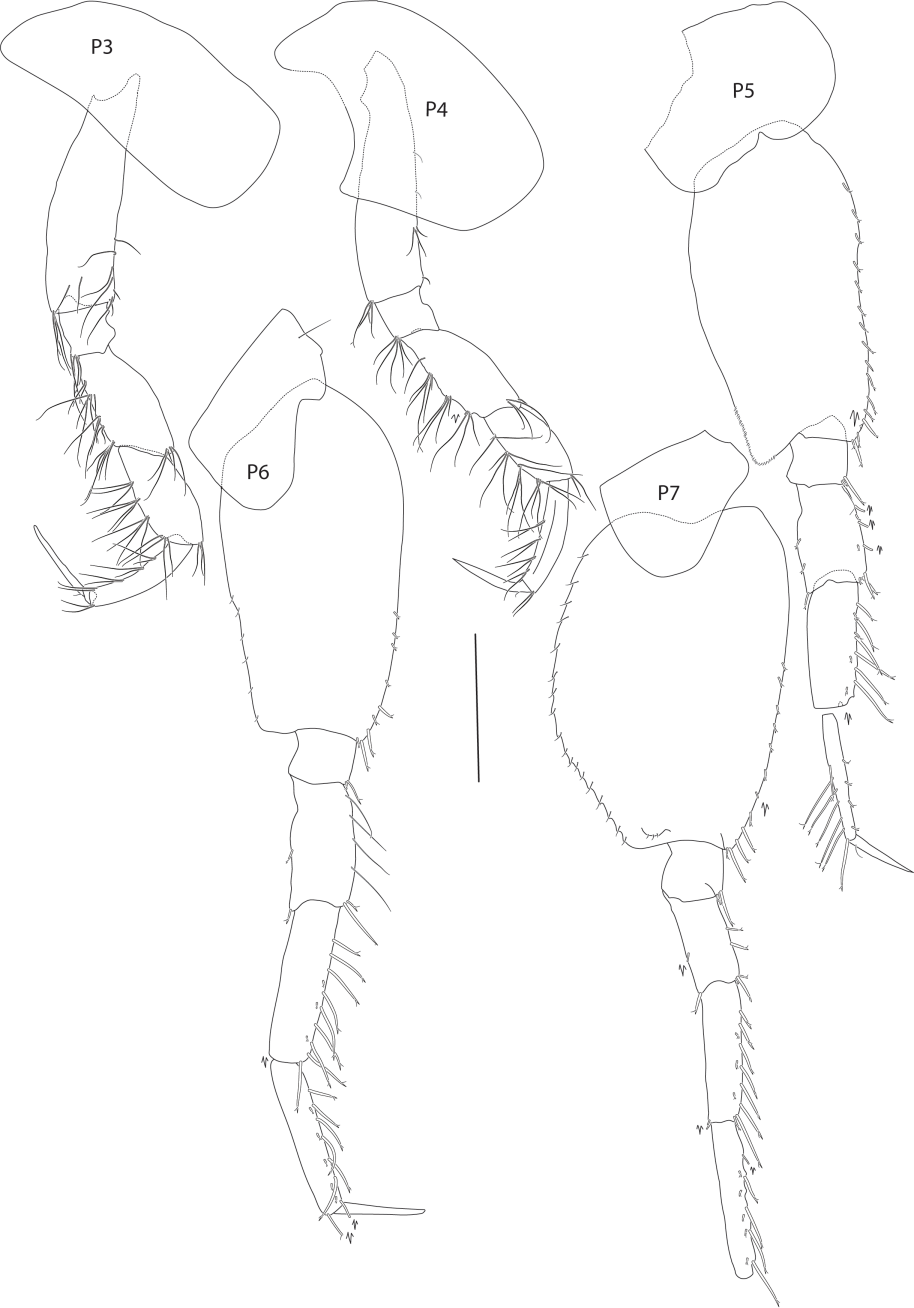
*Elimedon breviclunis* sp. nov., immature female, 12.8 mm, NHMUK 2025.10. Scale bars: 1 mm (P3–P7).

**Pereon: *Gnathopod 1*** (Fig. 2): coxa subrectangular, length 1.5 × width, anterior margin slightly convex, anterodorsal corner rounded, posterior margin nearly straight, distal margin straight; basis, anterior margin with short setae proximally and three long setae distally; ischium shorter than merus; carpus length 1.27 × propodus, posterior margin setose; propodus subchelate, subovate, palm acute, palmar corner defined by one medial and one lateral spine; dactylus narrow, overriding palm corner. ***Gnathopod 2*** (Fig. 2): coxa subrectangular, length 2.2 × width; basis narrow, length 3.8 × width, margins lacking setae; ischium longer than merus; carpus length 1.45 × propodus; propodus subchelate, subovate, with anterodistal groups of long pectinate setae, hind margin setose, palm short, nearly transverse; dactylus overriding palm corner. ***Pereopod 3*** (Fig. 4): coxa subrectangular, with anterior margin very slightly convex, posterior margin slightly concave, length 2.14 × width; merus slightly longer than carpus, posterior margins with clusters of long setae; propodus subequal in length to carpus, posterior margin setose; dactylus straight, shorter than propodus. ***Pereopod 4*** (Fig. 4): coxa length 1.4 × width, anterior margin convex, posterior margin deeply excavate proximally, with wide subtriangular, posterodistal lobe located at distal 52% of the coxa length, ventral margin straight; remaining pereopod articles as in pereopod 3. ***Pereopod 5*** (Fig. 4): coxa slightly posterolobate, length twice width; basis length twice width, anterior margin spinose, posterior margin slightly convex, with short, narrow posterodistal lobe extending slightly past half of ischium; merus weakly expanded, slightly shorter than carpus, anterior margin with four slender spines; carpus anterior margin with six slender spines; propodus narrow, length equal to carpus, anterior margin with seven slender spines; dactylus straight, shorter than propodus. ***Pereopod 6*** (Fig. 4): coxa strongly rounded, length twice width; basis length twice width, anterior margin slightly convex with small spines distally, posterior margin straight posterodistal lobe small and not extended distally; remaining pereopod articles as in pereopod 5 except merus-carpus slightly longer. ***Pereopod 7*** (Fig. 4): coxa subtriangular, posterolobate; basis broadly expanded, length 1.4 × width, anterior margin with weak spines distally, posterior margin convex, with small serrations and setules, posterodistal lobe not extending distally; merus-propodus as in pereopod 6 but narrower, anterior margins of merus-carpus with long slender spines; dactylus missing.

**Figure 5.**
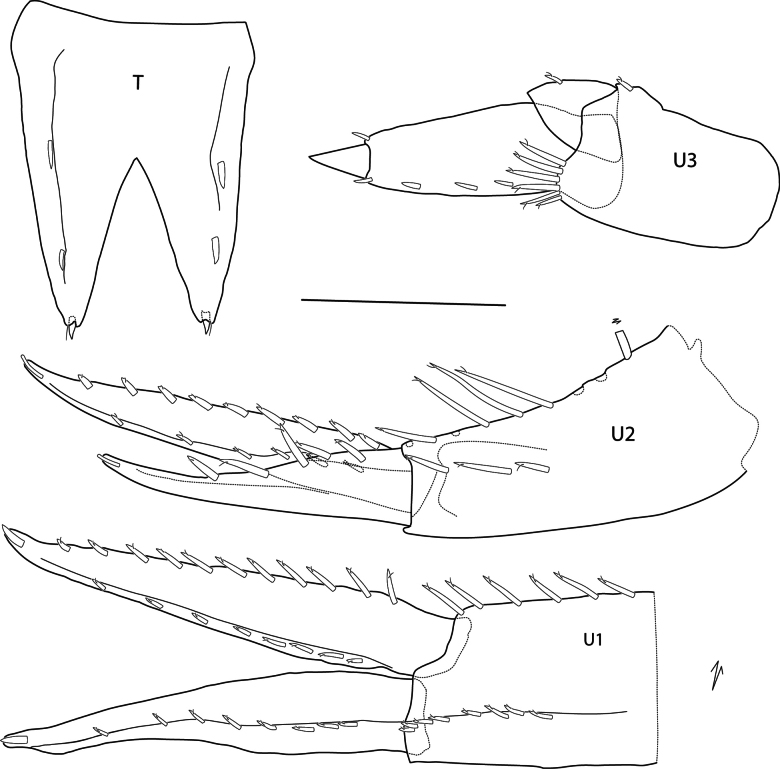
*Elimedon breviclunis* sp. nov., immature female, 12.8 mm, NHMUK 2025.10. Scale bar: 0.5 mm (U1–U3, T).

**Urosome** (Figs 1, 5): ***Uropod 1*** peduncle slightly longer than rami (partially broken and missing), dorsolateral and dorsomedial margins spinose, with > 8 and > 6 spines, respectively (peduncle broken); rami lanceolate, apically with a single inset spine, inner ramus slightly longer than outer, dorsolateral and dorsomedial margins with eight and 11 spines, respectively; outer ramus, dorsolateral margin with eight spines, dorsomedial margin lacking spines. ***Uropod 2*** peduncle subequal in length to outer ramus, dorsolateral and dorsomedial margins with three and nine slender spines, respectively, dorsomedial spines long; rami lanceolate with apical inset spine, inner ramus longer than outer ramus, dorsolateral and dorsomedial margins with nine and six slender spines, respectively; outer ramus dorsolateral margin with five spines. ***Uropod 3*** not extending past end of uropod 2, peduncle 0.86 × length of biarticulate outer ramus, with six distoventral spines; second article of outer ramus short, 0.2 × length of article 1, article 1 with four dorsolateral and one dorsomedial spine; inner ramus reduced, 0.3 × length of outer ramus, with one spine. ***Telson*** ~ 1.5 × longer than wide, widely cleft, and V-shaped, ~ 52%, lobes strongly tapering distally with two lateral, submarginal spines and one apical spine in middle of lobe tip.

**Oostegites** present on gnathopod 2 and pereopods 3–5, but non-setose, slender, largest on gnathopod 2 and pereopods 3 and 4, slightly smaller on pereopod 5.

**Gills** present on gnathopod 2 and pereopods 3–6, lacking on pereopod 7.

**Sexual dimorphism** (Fig. 1). Male antenna 2 flagellum more elongate, with 42 articles.

##### Etymology.

The species name *breviclunis* is from the Latin: *brevis*, (f) meaning short, and *clunis*, (f) meaning rump or buttocks, thus alluding to the distinctively shortened and stout ‘‘rump’’ area (pleon and urosome) of the amphipod.

##### Remarks.

The new species *Elimedon breviclunis* sp. nov. is more similar morphologically to *E. cristatus* J.L. Barnard. 1962, than it is to Ledoyer’s species *E. brevicaudatus. Elimedon breviclunis* sp. nov. has a telson which is quite long and deeply cleft, with widely separated lobes, in contrast to the much shortened and truncate telson of *E. brevicaudatus*. The uropod 3 inner ramus is very similar to *E. zabka* sp. nov., which is very shortened in contrast to *E. cristatus* which has an ordinary aequiramus condition of uropod 3. This has necessitated minor amendments to the genus diagnosis to account for the telson and uropod 3 inner ramus shortening which are variable among the four species now in the genus. A list of differences between the four species is provided in Table 2. These differences are minor but allow for the separation of the four species (for which a key is also provided).

**Table 2.** Differences in morphological character states between *Elimedon* species.

	***E. brevicaudatus***	***E. breviclunis* sp. nov**.	***E. cristatus***	***E. zabka* sp. nov**.
**Mandibular palp**	Article 3 very short, 0.5 × length of article 2	Article 3 short, 0.7 × length of article 2	Article 3 very short, 0.37 × length of article 2	Article 3 very short, 0.4 × length of article 2
**Gnathopod 1 carpus length**	Slightly longer: 1.34 × propodus	Slightly longer: 1.45 × propodus	Slightly longer:1.6 × propodus	Subequal to propodus
**Pereopods 5 and 6 bases**	P5 narrow, length 2 × width, P6 broader, length 1.5 × width	Both narrow: length 2 × width	Both narrow: length 1.9 × width	Both narrow: length 2.1 × width
**Uropod 3 inner ramus**	Shortened: length 0.6 × outer ramus	Strongly shortened: length 0.3 × outer ramus	Ordinary, aequiramus: length 0.95 × outer ramus	Shortened: length 0.56 × outer ramus
**Telson**	Short, length equal to width, rounded, cleft ~ 50%	Long, length 1.5 × width, widely cleft and V-shaped, ~ 52%	Long, length 1.87 × width, widely cleft and V-shaped, ~ 53%	Short, length equal to width, cleft and V-shaped, ~ 50%

**Distribution**. Abyssal Pacific Ocean, Clarion-Clipperton Zone, 4273–4299 m.

**Molecular data**. Sequence data for the holotype of *Elimedon breviclunis* sp. nov. is deposited in GenBank under accession numbers PV077112 (COI) and PV077021 (16S). Sequences of the paratype and additional individuals of the species are deposited in GenBank with the following accession numbers: PV077113–PV077115 (COI); PV077022–PV077024 (16S); PV077025 (18S); PV078006–PV078008 (H3); PV077026–PV077028 (28S). The species has also received a Barcode Index Number from Barcode of Life Data Systems: BOLD:AFV0648 (https://www.doi.org/10.5883/BOLD:AFV0648).

#### Elimedon zabka sp. nov.

Taxon classificationAnimaliaAmphipodaTryphosidae

17C89B07-DB9B-522D-A788-6DC825F07C8A

https://zoobank.org/7446943C-5E4D-4D1B-9BC1-5F691879C4C2

Figs 6, 7, 8, 9, 10

##### Type material.

***Holotype***: Pacific • immature female, 3.5 mm; one slide; Clarion-Clipperton Zone; 19.465°N, 120.025°W; depth 4026 m; 20/03/2015; Area of Particular Environment Interest No. 6 (APEI 6), RV "Thompson", Cruise ABYSSLINE-2, Station AB2-EB13, Epibenthic Sledge; SMF 63356; COI (PQ734642).

##### Type locality.

Clarion-Clipperton Zone, 19.465°N, 120.025°W; depth 4026 m.

##### Distribution.

Abyssal Pacific Ocean, Clarion-Clipperton Zone, 4026 m.

##### Diagnosis.

Mandibular palp article 3 very short, 0.4 × length of article 2; gnathopod 1 carpus subequal to propodus; Pereopods 5 and 6 bases both narrow, length 2.1 × width; uropod 3 inner ramus shortened, length 0.56 × outer ramus; telson short, length equal to width, cleft and V-shaped, ~ 50%.

##### Description.

Based on holotype immature female, 3.5 mm, SMF 63356.

**Figure 6.**
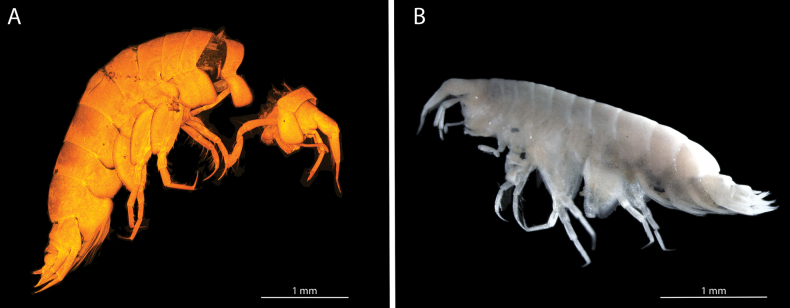
**A**CLSM photograph of *Elimedon zabka* sp. nov. **B** microscope photograph of *Elimedon zabka* sp. nov., habitus of the holotype immature female, 3.5 mm, SMF 63356. Photograph by Anna Jażdżewska. Scale bars: 1 mm.

**Figure 7.**
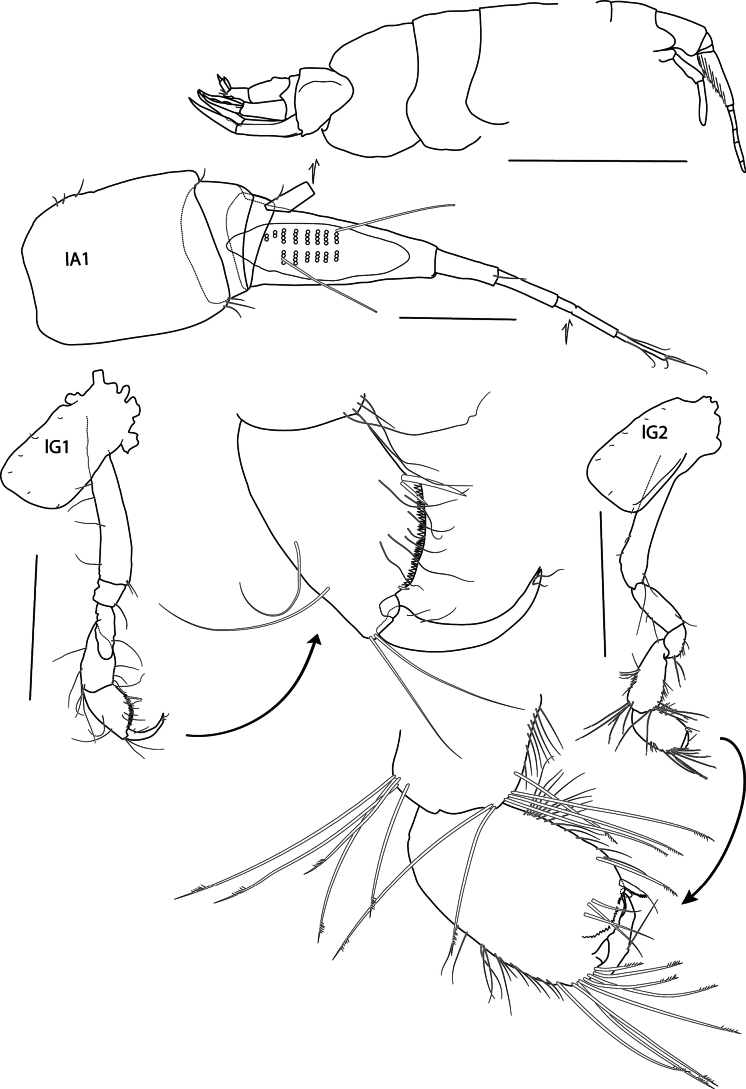
*Elimedon zabka* sp. nov., immature female, 3.5 mm, SMF 63356. Scale bars: 1 mm (habitus); 0.2 mm (A); 0.5 mm (G1, G2).

**Body** (Figs 6, 7): ***Pereonites 1*–*7*** (Fig. 6A) broader than deep. ***Pleonite 3*** (Fig. 6) with a rounded, posterodorsal elevation slightly overhanging urosomite 1. ***Urosomite 1*** (Fig. 7) with a weak, posterodorsal carina and lateral ridge. ***Urosomite 2*** (Fig. 7) short and not telescoped under urosomite 1. ***Urosomite 3*** (Fig. 6) with a slightly elevated, rounded boss. ***Epimeron 1*** (Fig. 7) quadrate, anterodistal corner rounded. ***Epimeron 2*** (Fig. 7) subquadrate, anterodistal corner rounded, posterior margin convex, posterodistal corner not produced, ventral margin straight. ***Epimeron 3*** (Fig. 7) anterodistal margin rounded, posterior margin convex, posterodistal corner produced into a small, upturned tooth, ventral margin slightly convex. ***Coxae 1*–*4*** (Fig. 6A) shorter than corresponding pereonites, coxae progressively longer, coxa 1 not shortened.

**Head** (Figs 6, 7): Slightly shorter than length of pereonites 1 and 2; rostrum short, not reaching end of lateral cephalic lobe. ***Lateral cephalic lobe*** (Fig. 7) broad, rounded apically. ***Eye*** not apparent in preserved specimen. ***Antenna 1*** (Fig. 7) short, length ¼ of body; peduncular article 1 dilated, length 1.24 × width [uncompressed measurement is 1.72 from Fig. 6A and 1.8 from Fig. 6B], dorsal keel very slight; peduncular articles 2 and 3 very short; flagellum four or five articulate, first article of flagellum callynophorate, length equal to peduncle 1, furnished medially with double row of aesthetascs; accessory flagellum broken, calceoli absent. ***Antenna 2*** likely slightly longer than antenna 1 (but flagellum missing), gland cone present; peduncular article 4 shorter than 5; flagellum broken off.

**Figure 8.**
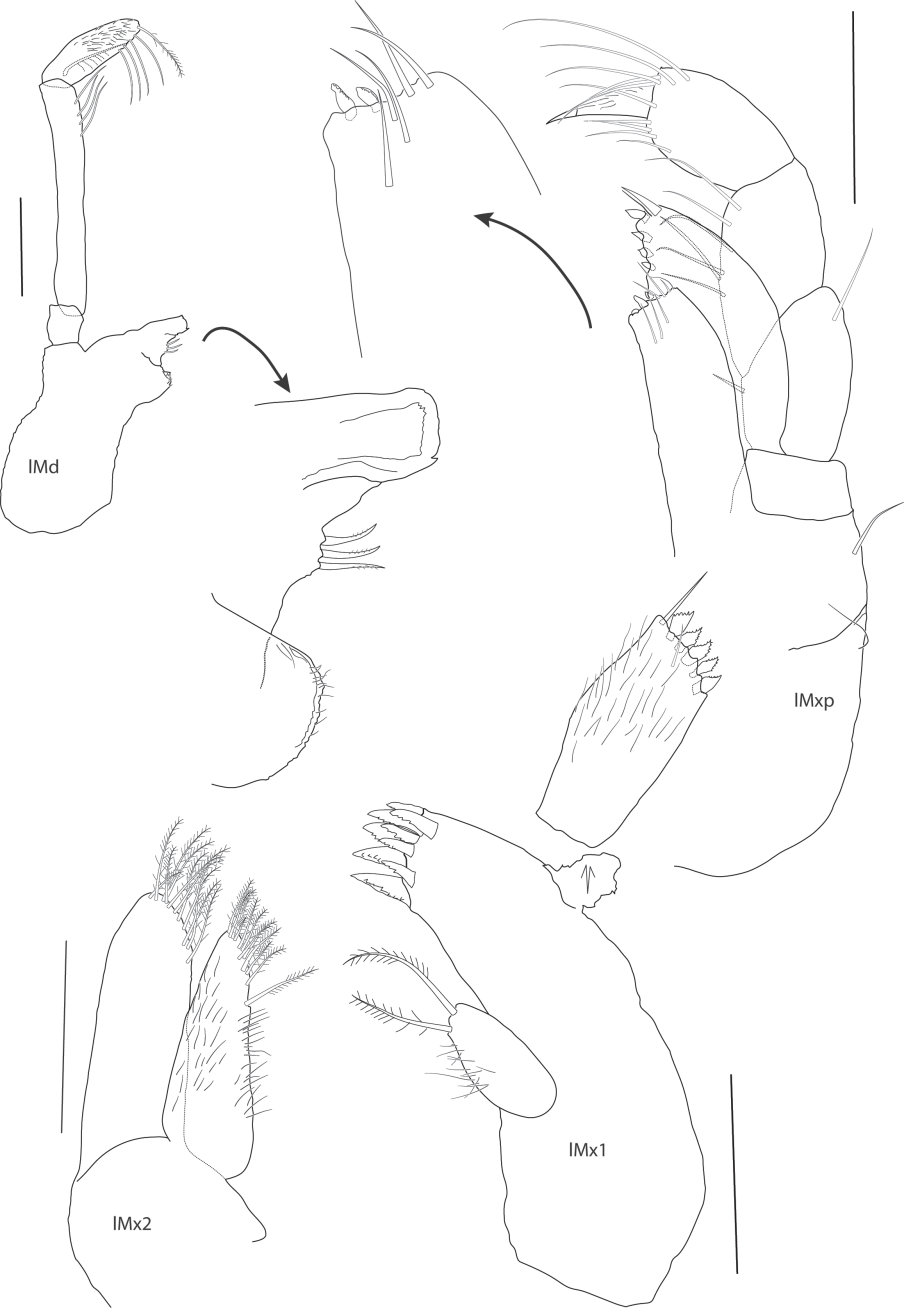
*Elimedon zabka* sp. nov., immature female, 3.5 mm, SMF 63356. Scale bars: 0.1 mm (Md, Mx2, Mx1, Mxp).

**Mouthparts. *Epistome*** (Fig. 6) approximately level with upper lip. ***Upper lip*** unknown, missing. ***Mandible*** (Fig. 8) incisor weakly convex, with tooth at posterodistal corner; left lacinia mobilis broad and finely serrate, accessory spine row with three small spines; molar lost during dissection; palp attached distal to molar, article 2 long, 2.55 × length of article 3, with four A2 setae, article 3 short, 0.4 × length of article 2, with a single A3 seta, four D3-pectinate setae, and one E3-seta. ***Lower lip*** unknown, missing. ***Maxilla 1*** (Fig. 8) inner plate narrowly ovate with two apical plumose setae; outer plate with nine spine-teeth in 5/4 crown arrangement; palp bi-articulate (damaged), article 2 not widened distally, with five contiguous conical apical spines, one distolateral and one facial seta. ***Maxilla 2*** (Fig. 8) inner plate slightly shorter and narrower than outer, tapering distally, both with rows of pectinate, apicomedial, marginal spines and setae, inner plate with one isolated plumose seta below. ***Maxilliped*** (Fig. 8) inner plate elongated, subrectangular, extending just past the distal end of the outer margin of palp article 1 and reaching 0.67 × length of outer plate, distal margin with two nodular spines and setae; outer plate narrowly subovate, length 2.0 × width, just reaching the distal end of palp article 2, with one strong distal spine and four medial nodular spines; palp weakly setose medially, articles subequal, article 4 slightly shorter than article 3.

**Figure 9.**
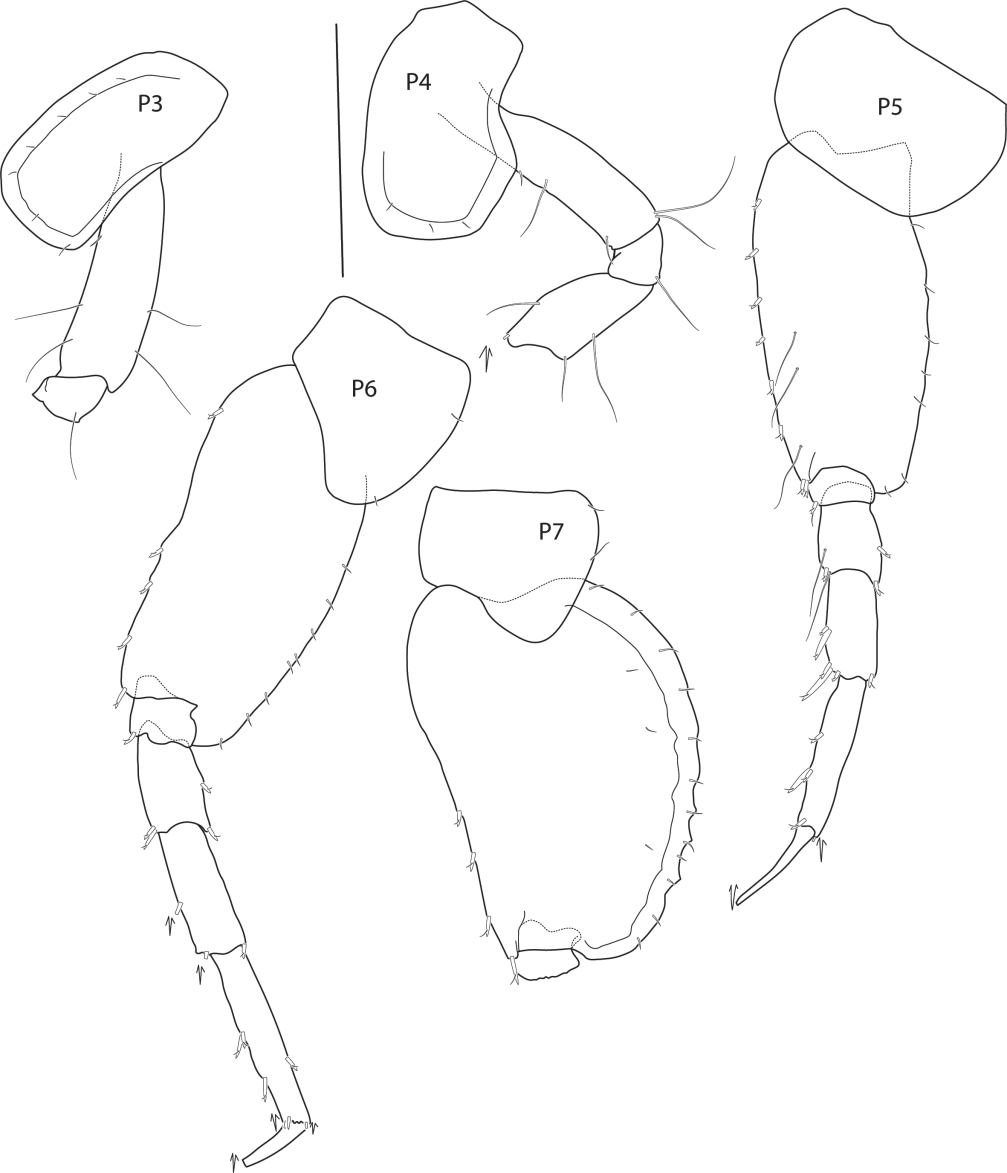
*Elimedon zabka* sp. nov., immature female, 3.5 mm, SMF 63356. Scale bars: 0.5 mm (P3–P7).

**Pereon. *Gnathopod 1*** (Fig. 7): coxa rectangular, length 1.6 × width, anterior margin very slightly convex, anterodorsal corner rounded, posterior margin slightly concave, distal margin straight; basis anterior margin with three or four medium setae; ischium shorter than merus; carpus subequal in length to propodus, posterior margin weakly setose; propodus subchelate, subrectangular, palm acute, convex, microserrate, palmar corner defined by two medial spines; dactylus narrow, strongly curved and reaching palm corner. ***Gnathopod 2*** (Fig. 7): coxa rectangular, length 1.9 × width; basis narrow, length 6 × width, margins with a few setae; ischium length 1.66 × merus; carpus length 1.25 × propodus, with cluster of long setae anterodistally and posterodistally; propodus subchelate, subovate, with anterodistal groups of long pectinate setae, hind margin setose, palm short, defined by one lateral and one medial spine, nearly transverse; dactylus reaching palm corner. ***Pereopod 3*** (Figs 6A, 9): coxa rectangular, with anterior margin very slightly convex, posterior margin slightly concave, length 2.0 × width; merus longer than carpus, posterior margins of both articles with a few long setae; propodus longer than carpus, posterior margin setose; dactylus long, curved, and slightly shorter than propodus. ***Pereopod 4*** (Figs 6A, 9): coxa length 1.5 × width, anterior margin convex, posterior margin excavate proximally, with broad, truncated posterodistal lobe located at distal 40% of the coxa length, ventral margin straight; remaining pereopod articles as in pereopod 3. ***Pereopod 5*** (Fig. 9): coxa broadly subovate, length 1.5 × width; basis, length 2.1 × width, anterior margin spinose, posterior margin slightly convex, with short, posterodistal lobe extending to half-length of ischium; merus short, weakly expanded, shorter than carpus, anterior margin with two long setae and one distal spine, posterior margin with two spines; carpus anterior margin with two large and three small spines; propodus narrow, length 1.5 × carpus length, anterior margin with four spines; dactylus straight, shorter than propodus. ***Pereopod 6*** (Fig. 9): coxa strongly posterolobate, rounded; basis length 2.1 × width, anterior margin sinuous with small spines distally, posterior margin convex with setules, posterodistal lobe extending to half-length of ischium; remaining pereopod articles as in pereopod 5 except merus-propodus 1.2 × longer. ***Pereopod 7*** (Figs 6, 9): coxa subovate, posterolobate; basis broadly expanded, length 1.36 × width, anterior margin sinuous with weak spines distally, posterior margin strongly convex with setules, with three prominent serrations distally, posterodistal lobe broad just reaching distal end of ischium; merus-dactylus as in pereopod 6 but slightly narrower.

**Figure 10.**
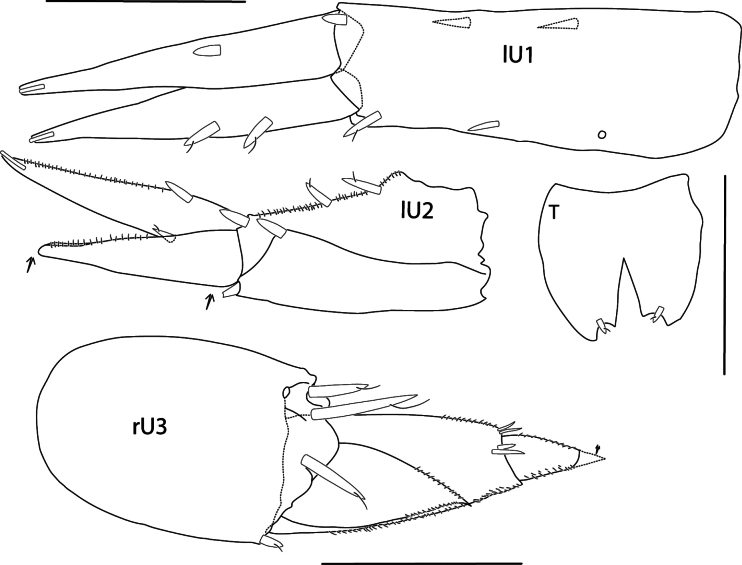
*Elimedon zabka* sp. nov., immature female, 3.5 mm, SMF XXXX. Scale bars: 0.2 mm (U1, U2, T); 0.1 mm (U3).

***Urosome*. *Uropod 1*** (Figs 6, 10): peduncle length 1.2 × rami, dorsolateral and dorsomedial margins weakly spinose; rami lanceolate, subequal, weakly spinose, apically with one inset spine. ***Uropod 2*** (Figs 6, 10): peduncle length 1.3 × outer ramus, dorsolateral and dorsomedial margins with three and one spines, respectively; rami lanceolate with apical inset spine, inner ramus longer than outer ramus, dorsolateral margin with one spine; outer ramus partially broken [regenerated]. ***Uropod 3*** (Figs 6, 10) not extending past end of uropod 2, peduncle subequal to length of biarticulate outer ramus, with three distoventral spines; second article of outer ramus 0.4 × length of article 1 [tip broken off], article 1 with two dorsolateral and two dorsomedial spines; inner ramus acute, reduced, length 0.56 × outer ramus. ***Telson*** (Figs 6, 10) short, length equal to width, lateral margins convex, cleft in a narrow V-shape, ~ 50%, lobes with prominent apicomedial incision inset with a single small spine.

##### Etymology.

The species name *Żabka* is a Polish word meaning “little frog” and is used as a noun in apposition. The name has been chosen to honour the Sustainable Seabed Knowledge Initiative funded taxonomic workshop at the University of Lodz, Poland, which was the first Amphipod Workshop which GVD attended, and as a reminder of the friendships that were forged in the cold, snowy Polish winter.

##### Remarks.

The new species *Elimedon zabka* sp. nov. is described from a single specimen of unknown sex, 3.5 mm in length. Like *E. breviclunis* sp. nov. the uropod 3 inner ramus is strongly shortened, which differentiates these two new species from both *E. cristatus* which has an ordinary aequiramus condition of uropod 3, and *E. brevicaudatus*, which presents an intermediate, slightly shortened inner ramus. The telson in *E. zabka* sp. nov. is shortened (length equal to width), truncate with convex lateral margins, and cleft in a narrow V-shape which is most like the shortened and truncate telson of *E. brevicaudatus*.

##### Distribution.

Abyssal Pacific Ocean, Clarion-Clipperton Zone, 4026 m.

##### Molecular data.

Sequence data for the holotype of *Elimedon zabka* sp. nov. is deposited in GenBank under accession number PQ734642. The species has also received a Barcode Index Number from Barcode of Life Data Systems: BOLD:AEB4890 (https://www.doi.org/10.5883/BOLD:AEB4890).

COI sequences of *Elimedon breviclunis* showed zero intraspecific genetic divergence. The interspecific divergence between *E. breviclunis* and *E. zabka* was high, ranging from 29–31%. This is unusual for two species within the same genus and further highlights the need for study of the relationships between *Hippomedon* and allied genera within the family Tryphosidae. The molecular sequences provided in this study will help in this process.

### Key to the species of *Elimedon*

**Table:** 

1	Uropod 3 inner ramus strongly shortened	**2**
–	Uropod 3 ordinary, aequiramus	***E. cristatus***
2	Telson longer than wide, lobes narrow, widely separated	***E. breviclunis* sp. nov**.
–	Telson length equal to width	**3**
3	Epimeron 3 posterodistal corner produced into a small, upturned tooth	***E. zabka* sp. nov**.
–	Epimeron 3 posterodistal corner broadly rounded, lacking tooth	***E. brevicaudatus***

## Supplementary Material

XML Treatment for Elimedon

XML Treatment for Elimedon breviclunis

XML Treatment for Elimedon zabka

## Additional information

### Author ORCIDs

Tammy Horton https://orcid.org/0000-0003-4250-1068

Georgina Valls Domedel https://orcid.org/0009-0005-8932-6641

Eva C. D. Stewart https://orcid.org/0000-0001-8383-5705

Ed A. Hendrycks https://orcid.org/0000-0001-6199-7121

### Data availability

All of the data that support the findings of this study are available in the main text.
